# Fungal Infections: Their Diagnosis and Treatment in Transplant Recipients

**DOI:** 10.1155/2012/106923

**Published:** 2012-08-26

**Authors:** David H. Van Thiel, Magdalena George, Christopher M. Moore

**Affiliations:** Section of Hepatology, Rush University Medical Center, Chicago, IL 60612, USA

## Abstract

Systemic fungal infections typically occur in individuals who are seriously ill with recognized risk factors such as those frequently found in transplant recipients. Unfortunately, they are often diagnosed late, when the efficacy of the available treatments is low, often less than 50%, and the cost in terms of lives lost, hospital length of stay, and total hospital costs is substantially increased. The application of antifungal therapies associated with reported efficacy rates greater than 50% are those used prophylactically. When used prophylactically, these infections are reduced in greater than 95% of the expected cases. The choice of a prophylactic agent should be based upon its ease of administration, lack of adverse effects, reduced likelihood of potential drug interactions, and its efficacy in patients with established risk factors and comorbid disease processes that include renal, hepatic, and chronic pulmonary disease. The indications for the use of currently available antifungal agents, their adverse effects, drug interactions, ease of dosing, and applicability in patients with preexisting disease states, and especially in liver transplant recipients, are presented in this paper.

## 1. Epidemiology

The frequency and variety of invasive fungal infections have increased greatly over the last three decades as a consequence of changes in medical and surgical care, particularly in intensive care units which utilize invasive catheters for monitoring, coupled with the use of more potent immunosuppression and antibiotic agents [1]. The current increase in invasive fungal infections is the result of changes in disease management with the use of powerful immunosuppressive agents, multiple antibiotics, the use of organ support procedures that include mechanical ventilation, hemodialysis and venovenous hemofiltration, and parental hyperalimentation. These medical and procedural advances coupled with the application of more aggressive antineoplastic therapies and transplantation of individuals with preexisting cardiopulmonary, renal, and hepatic disease processes have changed the frequency and approach to fungal infections. Specifically, as a direct result of these advances and therapeutic successes, the population at risk for fungal infections has expanded greatly [2, 3]. In the early 1980s, systemic candidiasis was recognized as an important medical problem. The mortality associated with candidiasis increased steadily until 1988, when it peaked at a rate of 0.6 per 100,000 per population [4]. As a result of recent advances in the treatment of invasive candidiasis, mortality stemming from Candidemia has decreased annually since its peak. Nonetheless, systemic candidiasis remains the fourth most common nosocomial bloodstream infection [3]. Although the number of bloodstream infections due to *C*. *albicans* has decreased, those due to other *Candida* species, particularly *C*. *glabrata*, *C*. *krusei*, and *C*. *parapsilosis*, have increased [1].

In contrast to candidiasis, infections and death as a result of other fungal pathogens, particularly *Aspergillus* species, have continued to increase since the late 1980s [4–9]. The mortality of invasive aspergillosis infections remains very high, particularly in transplant recipients despite the use of new diagnostic methods and advances in therapy [10].

The risk for systemic fungal infection is greatest in those with hematologic diseases requiring allogeneic hematopoietic stem cell transplantation followed by autologous grafts, other hematologic disorders associated with severe and prolonged leukopenia, those with solid organ neoplasms, and solid organ transplanted individuals [11–20]. The presence of confounding chronic pulmonary disease and heart/lung transplantation increases the risk for infections due to yeasts and molds, particularly *Aspergillus*.

The specific fungal and yeast pathogens experienced in hematopoietic stem cell transplantation and solid organ transplantation differ dramatically. Specifically, invasive *Aspergillus* and other molds account for 70% of the fungal/mold infections in hematopoietic stem cell recipients, while only a minority of solid organ transplant recipients acquire these infections unless they have confounding chronic pulmonary disease or the recipient is exposed to a hospital construction site or dust containing molds [10–13, 17, 18]. Nonmold infections in hematopoietic stem cell recipients account for 30% of the total fungal/mold infections in this population. In contrast, almost all of the infections in solid organ transplant recipients are due to fungal agents with only a minority occurring as a result of *Aspergillus* and other molds. In this later group, invasive candidiasis accounts for 50% of the total infections followed in order by *Cryptococcus* (7%), endemic mycosis (6%), and finally all other fungal or mold infections combined account for 37% of the total. These differences in the pattern of invasive fungal infections between hematopoietic stem cell transplant and solid organ transplant are, at least in part, due to the routine use of azole agents for prophylaxis in the former group, but not those in the latter.

## 2. Differences due to the Type of Organ Transplant

As expected, the overall incidence of systemic fungal infections is greatest in those receiving bone marrow transplants as compared to solid organ transplants. The incidence varies within each group as a function of the type of marrow transplanted and solid organ transplanted. In marrow recipients, the incidence is greatest in those receiving mismatched related and unrelated allogeneic stem cells (5.9%). Those receiving matched related allogeneic stem cells have a lower incidence of 3.7% and those receiving autologous stem cells have the lowest incidence (0.6%). These rates of infection reflect the major differences in chemoablative therapies used to condition the marrow, the duration of posttransplant cytopenia experienced, and the immunosuppression differences utilized between these various groups [17].

Differences in the incidence and type of systemic fungal infections occurring as a consequence of the particular organ transplanted are seen in solid organ transplant recipients as well. Those receiving lung grafts have the highest incidence (7.9%) followed by heart (3.4%), then liver (3.1%), renal (1.1%), and pancreas (0.7%). Those receiving lung and heart transplants have a greater incidence of *Aspergillus* infections while those receiving nonthoracic solid organs experience candidiasis as their major fungal pathogen [1–4, 17–19]. The risk for candidiasis in solid organ recipients is greatest early after transplantation (first 2-3 months after transplant) and then declines as other fungal pathogens including aspergillosis, histoplasmosis, coccidioidomycosis, and blastomycosis become more prominent, with variation depending on the geographic location of the recipient [2]. The early infections due to candidiasis are a result of the use of indwelling catheters, central lines, abdominal wounds, drains, and secondary operations as well as the use of parenteral nutrition and mechanical ventilation [5]. Late infections that occur months to years after transplantation are a consequence of the life-long immunosuppressive agents that these patients take to prevent rejection and the unique local environmental exposures the recipient experiences.

## 3. Cost of Fungal Infections

The direct costs of fungal infections are substantial [19]. The global cost of candidiasis is 2.5 times that for *Aspergillus* infections. However, when the global costis corrected for the number of individuals infected, the individual cost is 2-3-times greater for those with an *Aspergillus* infection as compared to that experienced by one with candidiasis [19].

## 4. Diagnosis of Fungal Infections

Early diagnosis and treatment are critically important in terms of obtaining a better outcome defined as a reduced morbidity and mortality. The diagnosis of invasive fungal infections is difficult because of the lack of specific signs and symptoms until late in the disease process and the difficulty associated with documenting a diagnosis with current diagnostic tools, obtaining infected tissue required to establish a specific diagnosis, and in some cases defining the isolated agent's sensitivity to the therapeutic regimen being utilized [20].

## 5. Therapeutic Definitions

The high mortality of invasive surgical infections is due in large measure to the delay in recognizing an infection in individuals at risk for severe infections and the difficulty in establishing an early diagnosis as a result of the nonspecific clinical features, low sensitivity of microscopic diagnostic methods, the difficulty in obtaining infected tissue for histologic and microbiologic diagnostic procedures, and appropriately interpreting imaging procedures [20].

As a direct consequence of these factors prophylactic, empiric, and preventative therapies have been developed and utilized. The specific antifungal agent chosen for each of these therapeutic approaches varies between centers and specific types of transplantation. Factors that affect the choice of antifungal agent include characteristics of the patient, the clinical circumstances, and the presence or absence of overt sepsis and/or hemodynamic instability.

Empiric therapy is defined as the initiation of antifungal treatment in an individual at high risk for an invasive fungal infection and manifesting symptoms and/or signs of an infection but without microbiological documentation of the infection. Empiric therapy utilizes a broad-spectrum antifungal agent for 3 or more days until deescalation is possible based upon the specific infection identified, its location, and the patient's clinical status.

Prophylactic therapy is defined as the use of an antifungal agent with intent to prevent the likelihood of an invasive fungal infection in an individual at high risk for such an infection. Preemptive therapy is defined as the initiation of antifungal therapy based upon the results of an early diagnostic test.

## 6. Diagnostic Tools

Currently available diagnostic tools for establishing a diagnosis of an invasive fungal infection include the following: galactomannan, (1,3)-*β*-glucan, and *C*. *albicans* germ tube antibody detection. Each of these procedures has its own set of problems that limit their widespread application.

The galactomannan assay is an enzymatic immunoassay which has been FDA approved and is used in the United States and Europe. The assay can detect galactomannan in blood 5–8 days on mean (range 1–27 days) before the onset of clinical signs and symptoms of an invasive fungal infection. It is a nonspecific test and only suggests the presence of a fungal infection as it measures a component of fungal hyphae. When positive, the level determined varies as a function of the infectious agent burdens and can be used to monitor the response to therapy [21–24]. Unfortunately, false-positive results occur in 5.7%–14.0% of adults and as high as 83% in neonates. The cause of these positive results is not entirely clear but the use of piperacillin-tazobactam in adults and cross-reactivity with antigens expressed in *Bifidobacterium* species in neonates have been suggested as possible causes [25, 26].

Overall, the galactomannan assay has moderate accuracy for the diagnosis of an invasive fungal infection in an immunocompromised individual and is more efficacious in those with hematologic malignancies or hematologic stem cell transplant procedures than in those undergoing solid organ transplant procedures [21–24]. Nonetheless, the presence of a positive galactomannan assay result utilizing bronchoaveolar lavage fluid in a solid organ transplant recipient with clinical signs of either a bronchial infection or pneumonia is highly diagnostic in this population [26, 27].

The (1,3)-*β*-d-glucan assay has been approved by the FDA also and when positive in blood suggests the presence of a fungal infection. It, like the galactomannan assay, is a broad-spectrum fungal marker that requires subsequent microbiological and imaging studies to define the specific infection. Its usefulness reflects the fact that glucans are a critical compound of the cell wall of most pathologic fungi except for *Cryptococcus* and zygomycetes [28–33].

A major problem with the (1,3)-*β*-d-glucan assay is its requirement for endotoxin and glucan-free glassware, and the presence of false positive result as a result of the presence of albumin, immunoglobulins, glucan-containing materials, gram-positive bacteria, and hemodialysis. As a result, its major usefulness is to exclude the possibility of a fungal infection [29–33].

Antibodies to *C*. *albicans* germ tube antigens have been useful at detecting invasive candidiasis due to a broad spectrum of *Candida* species [34, 35]. It has been most useful in the detection of *Candida* infections in drug users, hematologic cancer, and transplant recipients, as well as medical patients in an ICU setting [34, 35]. Its use in solid organ transplant recipients has not been evaluated.

The uses of polymerase chain reactions (PCR) to detect fungal DNAs are available in research laboratories but are not standardized or FDA approved. More bothersome is the fact that because of their sensitivity, they may be positive in samples obtained from patients with colonization rather than infection. Consequently, the diagnosis of an infection rather than colonization may in fact require the use of a less sensitive test for confirmation.

Microbiological cultures of biologic fluids and tissue for the detection of an invasive fungal infection require multiple days and occasionally weeks for the identification of a specific fungal pathogen. This said, they are highly specific and can be used for antifungal resistance testing if necessary.

## 7. Available Antifungal Agents (Tables 1 and 2)

In general, antifungal agents target components of the fungal cell wall that result in defective cell wall homeostasis and induce an osmotic stress that leads to lysis and fungal death. The polyenes (amphotericin) bind to ergosterol, the principle sterol component of the fungal cell membrane resulting in a loss of cell wall integrity. The azoles (fluconazole, itraconazole, voriconazole, and posaconazole) inhibit enzymes involved in ergosterol synthesis. The echinocandins inhibit glucan synthesis. Glucan is a long chain polymer responsible for fungal cell wall stability. It accounts for 30–60% of the cell wall mass in *Candida*, *Aspergillus*, and *Saccharomyces* species. Importantly, human cells do not contain glucan, thus accounting for the low rate of human toxicity associated with this class of agents.

### 7.1. Polyenes

Amphotericin has been the principal agent for the treatment of invasive fungal infections for more than half a century. Its efficacy is based upon its ability to bind to ergosterol, the principal sterol in the fungal cell wall, inducing a loss of cell wall osmotic regulation and lysis of the infecting fungus (fungicidal). The limiting factor for its use is nephrotoxicity, particularly a progressive renal dysfunction associated with hypokalemia, renal tubular acidosis, and hypocalcemia. Lipid-based amphotericin preparations have attenuated the nephrotoxicity compared to the original agent but continue to have a similar pattern of adverse effects. Unfortunately, these lipid-based amphotericin preparations do not appear to have greater efficacy and are considerably more expensive than the original material.

The use of predosing hydration regimens with normal saline and a continuous infusion of amphotericin has reduced the fever, chills, and flushing associated with its use, but can be problematic in transplant patients with preexisting renal and/or hepatic dysfunction, who often are volume overloaded complicated further by a low serum albumin level [36]. These same individuals can occasionally experience an acute pulmonary reaction similar to pulmonary edema while receiving amphotericin.

Amphotericin is an accepted antifungal agent for *C*. *albicans* but has reduced activity against *C*. *glabrata*, *C*. *krusei*, *C*. *lusitaniae*, and molds. Moreover, polyenes have not been shown to be of any value in prophylaxis. The toxicity of the polyenes, especially their nephrotoxicity and their expense (lipid solubilized polyenes), make them less likely to be used than other currently available agents.

### 7.2. Azoles

This class of agents is less toxic than the polyenes and can be administered both orally and intravenously. They act by inhibiting ergosterol synthesis and through other unidentified mechanisms. Their perturbation of a large number of P450 enzyme systems limits their use in individuals, who require other agents which are metabolized by P450 enzymes which may mandate an alternative dosing regimen of these other agents (see Tables 3(a) and 3(b)). The superior toxicity profile of fluconazole, its availability in an intravenous and oral formulation, and its low cost make it the agent of choice for hemodynamically stable patients with Candidemia.

The activity of voriconazole against *Candida* is superior to that achieved with fluconazole based upon MIC data, its activity against fluconazole-resistant species, and its wider spectrum make it the preferred agent for hemodynamically unstable patients or those where the infection is due to a nonalbicans *Candida*, *Aspergillus*, or any other mold [37]. It, however, has a clinically important effect on the metabolism of calcineurin inhibiting agent (immunosuppressive agents), resulting in a marked increase in their whole blood levels, which can reach toxic levels unless the dose of these immunosuppressive agents is reduced markedly. Moreover, two unique toxicities have been associated with the use of voriconazole. These are the development of a visual disturbance and cutaneous photosensitivity. The visual disturbance occurs in as many as 45% of individuals receiving the agent. Typically, it is transient and resolves with continued treatment. The cutaneous photosensitivity reaction is unusual and importantly is not prevented with sun-screen lotions. It is fully reversible with drug discontinuation. The use of voriconazole and posaconazole is contraindicated when sirolimus is being utilized as part of the immune suppressive regimen (see Table 3(a)). Itraconazole can cause a unique complex of adverse effects consisting of hypotension, hypokalemia, and edema. A negative inotropic effect causing congestive heart failure has been identified as well and limits its usefulness in individuals with preexisting heart disease [38]. Itraconazole has two other limiting issues particularly in hematopoietic stem-cell transplant patients: its potential for hepatotoxicity and its reduced absorption when used in combination with either H_2_ blockers or proton-pump inhibiting agents.

### 7.3. Fluorocytosine

Fluorocytosine is a pyrimidine analog that inhibits both DNA and protein synthesis. Its principal use is in combination with other agents for the treatment of cryptococcal infections [39–41].The development of rapid drug resistance and its toxicity pattern (see Table 3(b)) limits its usefulness in other fungal infections [41].

### 7.4. Echinocandins

The echinocandins are semisynthetic lipopeptides that were isolated originally from various fungal agents and subsequently modified. Specifically, they are cyclic hexapeptides with an N-linked acyl-side chain that appears to be essential for their antifungal activity. They have different molecular weights that vary around 1200 daltons. This class of agents inhibits glucan synthesis, a major component of the fungal cell wall required for stability, especially in *Candida* and *Aspergillus* species. The echinocandins have been shown to enhance phagocytic activity of macrophages, an action that may also contribute to their efficacy in eliminating fungal infections. Moreover, they also have activity against preformed *Candida* biofilms and thus prevent *Candida* species from adhering to endothelial cells.

The pharmacokinetic characteristics of the echinocandins are shown in Table 5. There are very minor differences between the various agents except for the precise mechanism of their metabolism. Importantly, no dose adjustments have to be made for patients with renal disease. Currently, anidulafungin is the only available echinocandin that does not require a dose adjustment in cases with moderate liver disease defined as those having Child-Pugh scores between 7 and 9.

Because the echinocandins do not perpetuate cytochrome P450 enzyme systems and they do not interact with P-glycoprotein, as some azoles do, they do not affect the levels of the calcineurin-inhibiting immunosuppressive agents and the many other drugs used to manage other infections, hypertension, and cardiac arrhythmias frequently seen in transplant recipients (Tables 3(a) and 3(b)). Of particular interest to the transplant population, echinocandins have limited theoretical activity against *Pneumocystis carinii* (*P*. *jiroveci*) infections [42, 43].

They are efficacious also against the histoplasmosis species, blastomyces species, and coccidioides species—fungal agents that cause late onset infections in transplant populations.

Resistance to echinocandins has been reported [44, 45]. Unfortunately, available assessments of MIC values for the echinocandins do not clearly distinguish between sensitive and resistant fungi [37, 45–48]. As a result, these assays have to be interpreted with caution and in context.

The specific mechanisms responsible for resistance to the echinocandins are not clear but appear to be related to mutations in a subunit of glucan synthetase [44, 49]. Other minor mechanisms have been identified as well.

Elevations of serum transaminase and alkaline phosphatase levels are among the most common laboratory changes associated with caspofungin [50, 51]. Micafungin has recently had its use in patients with liver disease restricted (as noted in its new package insert) because of reports of acute hepatitis and hepatic failure occurring with its application [51]. The use of drugs such as rifampin, phenytoin, carbamezapine, efavirenz, and nevirapine causes a reduction in caspofungin levels and necessitate a 50% increase its dosage. Caspofungin reduces the AUC for tacrolimus by 20%, while cyclosporine has been reported to cause a 35% increase in the AUC for caspofungin. Micafungin increases the AUC for sirolimus by 21% and for nifedipine by 18%. These interactions have not been reported to occur with anidulafungin (Tables 3(a) and 3(b)).

It should be noted that the average wholesale cost of a 20-day course of caspofungin in the United States is approximately $7-8,000 as compared to $3-4,000 for anidulafungin and $2–5,000 for intravenous fluconazole (depending upon the dose). The average wholesale cost for micafungin for the same period would be $4-5,000.

## 8. Clinical Use of the Available Antifungal Agents

The agents available for treatment of invasive fungal infections are presented in Tables 1 and 2.

The mechanisms of action of each class of drugs, the specific disease indication for each drug, and the doses that are recommended for each indication are indicated in Table 1.

Table 2 presents data relative to the choice of antifungal therapy for each fungal agent that is currently approved by the FDA. The drug choices are indicated as first line (recommended), second line (less frequently utilized but effective), and third line (potentially having efficacy) and those having unknown efficacy.

Table 3 presents reported efficacy data and overall mortality data achieved when treating systemic fungal infections.

Voriconazole is currently the first line therapy for *Aspergillus* infections [52, 53]. Voriconazole is also the only agent indicated for infections due to *Fusarium* species and *Scedosporium apiospermum*. Only voriconazole, fluconazole, and itraconazole are available in oral as well as the intravenous formulations. The echinocandins have replaced azole agents for the treatment of invasive candidiasis [52–54].

The principal adverse effects and drug interactions of each antifungal agent are reported in Tables 3(a) and 3(b).

Table 6 lists the contraindications and FDA warnings for the available antifungal agents.

The effect of preexisting renal and/or hepatic dysfunction on the dosing of these available antifungal agents is shown in Tables 6(a) and 6(b). The dose of the azole agents needs to be reduced as the creatinine clearance declines. The effect of preexisting renal disease is inconsequential for the echinocandins. As noted, little data exists for the use of antifungal agents in individuals with hepatic disease. The only agent for which no dose adjustment is required for advanced hepatic disease is anidulafungin [55].

There are four emerging antifungal agents [47]. These include isavuconazole and ravuconazole both of which have a broad spectrum of activity, a large volume of distribution, and very long half-lives. Albaconazole has a broad spectrum of activity against *Candida* species, *Aspergillus* species, and *Cryptococcus* species as well as a long half-life. Aminocandin has a very long half-life enabling the drug to be given intravenously less often than daily.

Combination antifungal therapy is occasionally used in severe and clinically drug-resistant infections in an effort to maximize efficacy and potentially minimize toxicity [56, 57]. In selecting agents to be used in combination, only those that have different mechanisms of actions should be combined. Thus, the use of an agent acting at the cytoplasmic membrane (a polyene or an azole) plus an agent acting on DNA or protein synthesis (fluorocytosine) or a cell wall active agent (echinocandins) is recommended if combination therapy is to be utilized. It should be noted, however, that there are no definitive data supporting the use of combination therapy in the treatment of fungal infections *per se* but such can be reasonably implied from the use of combination treatments as an accepted treatment for bacterial and viral infections. This being said, considering the low efficacy rates reported for single agent treatment of invasive fungal infections in general, combination therapy has the potential to increase the efficacy of treatment in difficult-to-treat situations. A single study consists of a triazole and an echinocandin in solid organ transplant recipients. It shows a reduction in mortality in individuals with renal failure and invasive *Aspergillus* [56].

## 9. Clinical and Economic Relevance of Fungal Infections, Particularly Candidiasis

In the past, systemic fungal infections have been considered to be a problem only for neutropenic patients. However, beyond the risk factor of neutropenia, more recent data suggests that half of all hospital-acquired fungal infections have occurred in critically-ill surgical patients.

*Candida* species account for greater than 80% of all fungal nosocomial isolates [62–64] unlike *Aspergillus* species and the less common *Fusarium* and *Rhizopus* species which comprise only 10% of the remaining nosocomial isolates. Invasive candidiasis is the most frequently occurring invasive fungal infection and occurs most commonly in immunocompromised solid organ transplant recipients, those receiving chemotherapy, and those having multiple, complex abdominal surgical procedures.

As stated earlier, *Candida* species have become the fourth most common nosocomial bloodstream isolate, exceeded only by coagulase negative *Staphylococcus*, *Staphylococcus aureus*, and *Enterococci*. This fact is particularly important when it is recognized that less than half of these cases with invasive Candidemia documented at autopsy have had a positive premortem blood culture for *Candida* [59].

Invasive *Candida* infections have a mortality rate averaging between 25 and 38%. The specific *Candida* species accounting for Candidemia in high-risk populations have shifted over the last decade from *C*. *albicans* to more nonalbicans species, with approximately half the reported cases being due to the nonalbicans species [5]. More importantly these nonalbicans species (*C*. *glabrata*, *C*. *krusei*, and *C*. *parapsilosis*) have a greater mortality rate, account for the greater length of stay in ICUs, and are associated with greater rates of renal failure, thrombocytopenia, malignancy, and mechanical ventilation. The risk factors recognized for Candidemia in general include complicated abdominal operations, second operations, parenteral nutrition, the use of broad-spectrum antibiotics, the use of multiple vascular catheters, prior recognized *Candida* colonization, mechanical ventilation, and renal replacement therapy [58, 59].

In a prospective clinical trial examining the risk factors for *Candida* bloodstream infections in more than 4,000 surgical patients, those identified included previous surgery (RR = 7.3), acute renal failure (RR = 4.2), parenteral nutrition (RR = 3.6), and the presence of a triple lumen catheter (RR = 5.4) [58]. Other risk factors identified in other studies included ICU hospitalization >4 days, diabetes mellitus, HIV infections, central lines, neutropenia, chemotherapy, cancer (especially hematologic cancers), use of broad-spectrum antibiotics, the use of 3 or more antibiotics, and mechanical ventilation >2 days.

The initial response to a suspected Candidemia is to institute antifungal therapy with either voriconazole or an echinocandin and the removal of all vascular lines. It is important to recognize that blood cultures are positive in cases of invasive Candidemia in less than 50% of the time. Invasive *Fusarium* infections, similar to *Candida* infections, are detectable with blood culture in less than half of the cases. Worse invasive *Aspergillus* infections are rarely identifiable with blood cultures.

First line therapy for Candidemia remains controversial as studies have reported similar efficacy rates with amphotericin, fluconazole, echinocandins, and voriconazole [65–69]. With the increasing frequency of nonalbicans species especially in critically-ill patients the use of a broad-spectrum agent such as voriconazole, an echinocandin, or amphotericin may be more appropriate at least until the specific *Candida* species is identified to avoid the increased mortality occurring in cases wherein an inappropriate therapeutic agent is initially started. The limitation of intravenous voriconazole is its formulation with cyclodextrin which accumulates in individuals with impaired renal function. The many adverse effects of amphotericin identified earlier limit its use. Among the echinocandins, only anidulafungin has shown superiority over fluconazole [55]. Moreover, its efficiency, safety, and lack of cytochrome P450 metabolism suggest that it should be considered as a first line option for invasive candidiasis infection. Regardless of the choice of a specific echinocandin over fluconazole, echinocandins are recommended for use in individuals who are either critically ill or hemodynamically unstable.

Candidemia is associated with an increased cost of hospitalization estimated at $68,311 (95% CI $57,513–$79,108) and longer length of stay estimated at 23.1 days of hospitalization (95% CI 19.3–26.8 days) as compared to that of a DRG identified control population without Candidemia [70–72].

The incidence of invasive fungal infections in solid organ transplant recipients ranges from 5–42% [73]. Depending upon the organ being transplanted, being lowest for pancreas recipients and greatest for liver graft recipients.*Candida* species, and to a lesser degree *Aspergillus*, account for the vast majority of invasive fungal agents in solid organ transplant recipients [14]. *Cryptococcus* and endemic mycoses occur late, typically a year or more after transplantation.

Currently, most liver transplant centers use antifungal prophylaxis in the early postoperative period in individual recipients having either a complicated or repetitive posttransplant surgical procedures [74–76]. The principal problem associated with the use of azole therapy in transplant recipients is the interaction with calcineurin inhibitor agents that consequently requires a dose adjustment in one or the other agents.

## 10. Experience with Antifungal Agents in Individuals with Liver Disease

Although considerable data exists relative to the use and precautions to be utilized with antifungal agents in individuals with advanced renal disease, little data exist for those with advanced liver disease (Tables 6(a) and 6(b)).

At Barnes Jewish Hospital, anidulafungin has recently been utilized instead of the hospital preferred agent caspofungin in two specific disease categories with clinical efficacy [55]. The subjects in this report consisted of those with hepatic dysfunction (71% of the group) and those with potential drug interactions with caspofungin (21% of the group). These two groups were not mutually exclusive however with 4 patients with both criteria. Together, the two groups accounted for 83% of those receiving anidulafungin in the report.

The authors noted in their discussion that neither caspofungin nor micafungin have been studied in patients with severe liver disease and that anidulafungin is the only agent with suitable pharmacokinetic properties making it an acceptable agent in the liver disease population. In fact, 5 of the 35 patients (14.5%) in their study had a total bilirubin level greater than 10 mg/dL with two having a total bilirubin greater than 40 mg/dL. One of their patients had a transaminase level greater than 10 times the upper level of normal at the time when anidulafungin therapy was initiated.

Fungal infections following liver transplantation although infrequently increase the mortality rate and increase the overall cost of the perioperative transplant care [70–73]. The incidence of drug-resistant *C*. *albicans*, nonalbicans *Candida* species, *Aspergillus*, and other invasive molds in transplant recipients is increasing and is associated with a reduced survival rate [77, 78]. Risk factors for fungal infections following liver transplantation are well recognized and include preoperative renal failure, fulminant hepatic failure, prolonged preoperative hospitalization particularly in an intensive care unit, an excessive intraoperative transfusion requirement, early retransplantation or complications requiring a return to the operative theater, and the number of reoperations [73, 75, 77].

Despite this information, fungal prophylaxis has been a topic of considerable debate in terms of its efficacy, costs, choice of agents, the dose regimen, and the duration of therapy. Recently, failure to provide prophylaxis to a high-risk population of liver transplant recipients was associated with a 4-fold greater risk of fungal infections (*P* < 0.05) compared to an amphotericin prophylactic risk group [79–81].

Only a handful of well-designed prophylactic studies in liver transplant patients have been performed [82–88]. One study was inconclusive while two showed efficacy with fluconazoles in preventing invasive candidiasis. In particular, one study showed that fluconazole prevented infections due to *C*. *albicans* but not *C*. *glabrata* or *C*. *krusei* infections. A single report of the use of itraconazole used prophylactically posttransplant reduced the rate of fungal infections from a control value of 24% to 4% [86].

A meta-analysis of the use of antifungal prophylaxis in liver transplant recipients concluded that prophylaxis reduced the total number of episodes of infections as well as the morbidity directly related to the fungal infection but did not affect overall mortality [74].

No prophylactic studies in solid organ transplant recipients have demonstrated a clear beneficial effect at preventing invasive *Aspergillus*, but have been interpreted as suggesting such might be the case [75–77].

In contrast, studies of targeted therapy after liver transplantation have demonstrated voriconazole as the initial therapy of choice for invasive *Aspergillus*. Continuous infusions of amphotericin have also been shown to be effective and generally safe, but the many potential effects of amphotericin make voriconazole the preferred agent for this indication [36, 52].

## 11. Summary

1. Fungal infections, especially candidiasis and *Aspergillus*, are major health problems in seriously ill patients such as transplant recipients, despite the fact that the risk factors for such infections are well recognized and predictable.
2. Systemic fungal infections are typically diagnosed late and treatment is costly in terms of lives lost, hospital length of stay, and overall medical costs.
3. Despite a plethora of agents currently available for the treatment of systemic fungal infections, the efficacy of such treatments is low.
4. Anticipatory or prophylactic therapy of fungal infections in individual patients with 3 or more risk factors for such infections would appear to be prudent and may be the only way that these infections can be prevented. Clearly, further study is necessary to determine the best agent, optimal dosing, and duration of therapy in immunosuppressed and transplant patients.
5. Finally, as these agents are typically used in transplant patients with preexisting disease states, who are on a host of other therapeutic agents for comorbid disease states involving either the kidneys or the liver or both organs, the choice of the antifungal agent to be used in a particular case should be (a) the likelihood of therapeutic benefit, (b) the recognized and anticipated adverse events associated with their use, (c) the potential for drug-drug interactions, and, finally, their ease of administration with particular attention to their use in individuals with preexisting or concurrent renal and/or hepatic disease.

## Figures and Tables

**Table 1:** Antifungal agents currently available, mechanism of action, principle indications, and dosing schedule.

Class	Agents	Indication	Dosing schedule
Polyenes		Polyenes bind to sterols, preferentially to the primary fungal cell membrane sterol, and ergosterol. This binding disrupts osmotic integrity of the fungal membrane	

	(1) Amphotericin B	Invasive fungal infections include: aspergillosis, cryptococcosis, North American blastomycosis, systemic candidiasis, coccidioidomycosis, histoplasmosis, zygomycosis, *Conidiobolus*, *Basidiobolus*, sporotrichosis	0.3–1.5 mg/kg

	(2) AmBisome	Empirical therapy in febrile neutropenic pts.	3 mg/kg/day
		Cryptococcal meningitis in HIV pts.	6 mg/kg/day
		Visceral leishmaniasis	3-4 mg/kg/day

	(3) Amphotericin B Colloidal dispersion (ABCD)	No primary indication; only salvage therapy	3–6 mg/kg

	(4) Abelcet (ABLC)	No primary indication only salvage	5 mg/kg

Azoles		Azoles inhibit cytochrome P450 14a demethylase (P45014DM) required for ergosterol synthesis	

	(1) Voriconazole	Invasive aspergillosis and candidemia in nonneutropenic patients	6 mg/kg IV twice a day on day 1, then 4 mg/kg twice at day or 200 mg PO twice a day
		Esophageal candidiasis	200 mg PO twice a day given 1 hour before or after a meal

	(2) Fluconazole	Prophylaxis in transplant pts, invasive candida infections	800 mg/day IV on day 1, then 400 mg/day
		Oropharyngeal and esophageal candidiasis	200 mg/day IV or PO on day 1, then 100 mg/day
		Cryptococcal meningitis	200 up to 400 mg/day IV or PO

	(3) Itraconazole	Empirical therapy in febrile neutropenic patients, blastomycosis, pulmonary and extrapulmonary, histoplasmosis, chronic pulmonary and disseminated, and nonmeningeal histoplasmosis	200 mg IV twice a day or 100–400 mg po

	(4) Posaconazole	Prophylaxis of invasive *Aspergillus* and *Candida* in immunocompromised patients	200 mg (5 mL) three times a day with a full meal or nutritional supplement
		Oropharyngeal candidiasis	100 mg (2.5 mL) twice a day on day 1, then 100 mg (2.5 mL) once a day with a full meal or nutritional supplement

Echinocandins		The glucan synthesis inhibitors block fungal cell wall synthesis by inhibiting the enzyme 1,3-beta glucan synthase	

	(1) Anidulafungin	Candidemia, acute disseminated candidiasis	200 mg/day on day 1 then 100 mg/day
		Esophageal candidiasis	100 mg on day 1, then 50 mg/day

	(2) Caspofungin	Candidemia, acute disseminated candidiasis, empirical therapy in febrile neutropenic patients, and invasive aspergillus refractory to other therapies	70 mg/day on day 1, then 50 mg/day
		Esophageal candidiasis	50 mg/day

	(3) Micafungin	Candidemia, acute disseminated candidiasis	100 mg/day
		Esophageal candidiasis	150 mg/day
		Prophylaxis of Candida infection in pt undergoing HSCT	50 mg/day

**Table 2:** Pathogenic fungi and the antifungal agents *in vitro* activity**.

Microorganism	Antifungal agents				
	Fluconazole	Voriconazole	Posaconazole	Echinocandin	Polyenes
*Candida albicans*	1st line	1st line	1st line	1st line	1st line
*Candida glabrata*	Unknown	3rd line	3rd line	1st line	2nd line
*Candida tropicalis*	1st line	1st line	1st line	1st line	1st line

*Candida parapsilosis*	1st line	1st line	1st line	2nd line	1st line
*Candida krusei*	No activity	2nd line	2nd line	1st line	2nd line
*Candida guilliermondii*	1st line	1st line	1st line	2nd line	2nd line

*Candida lusitaniae*	3rd line	2nd line	2nd line	2nd line	2nd line
*Cryptococcus neoformans*	1st line	1st line	1st line	No activity	1st line
*Aspergillus fumigatus*	No activity	1st line	1st line	2nd line	2nd line
*Aserpgillus flavus*	No activity	1st line	1st line	2nd line	2nd line

*Aspergillus terreus*	No activity	1st line	1st line	2nd line	No activity
*Fusarium* sp.	No activity	2nd line	2nd line	No activity	2nd line
*Scedosporium apiospermum*	No activity	1st line	1st line	Unknown	Unknown

*Scedosporium prolificans*	No activity	Unknown	Unknown	No activity	Unknown
*Trichosporon* spp.	Unknown	2nd line	2nd line	No activity	3rd line
*Zygomycetes* (e.g., *Absidia*, *Mucor*, and *Rhizopus*)	No activity	No activity	1st line	No activity	1st line
*Dematiaceous molds * (e.g., *Alternaria*, *Bipolaris*, *Curvularia*, and *Exophiala*)	Unknown	1st line	1st line	3rd line	3rd line

Dimorphic Fungi					
* Blastomyces dermatitidis*	3rd line	2nd line	2nd line	No activity	1st line
* Coccidioides immitis*	1st line	2nd line	2nd line	No activity	1st line
* Histoplasma capsulatum*	3rd line	2nd line	2nd line	No activity	1st line
* Sporothrix schenckii*	3rd line	2nd line	2nd line	No activity	1st line

**Echinocandins, voriconazole, posaconazole, and polyenes have poor urine penetration.

**Table:** (a)

Drug A					Drug B		
Interacting drug	Echinocandins			Azoles			Polyenes
	Anidulafungin	Caspofungin	Micafungin	Fluconazole	Voriconazole	Posaconazole	Amphotericin B and lipid formulations
Amitriptyline				Inc A			
Aminoglycosides							Inc nephrotoxicity
Antineoplastic drugs							Inc nephrotoxicity
Astemizole							
Calcium channel blockers				Inc A	Inc A		
Carbamazepine		Dec B			Contraindicated Dec B		
Cidofovir							Inc nephrotoxicity
Cimetidine				Dec B			
Cisapride				Contraindicated	Contraindicated	Contraindicated	
Cyclosporine	Slight Inc B	Warning Inc B		Inc A, Inc nephrotoxicity	Inc A, Inc nephrotoxicity	Inc A, Inc nephrotoxicity	Inc nephrotoxicity
Dexamethasone		Dec B					
Didanosine							
Digitalis						Inc A	Drug interaction
Efavirenz		Dec B			Dec B, Inc A		
Ergot Alkaloids					Contraindicated	Contraindicated	
Flucytosine							Drug interaction
Foscarnet							Inc nephrotoxicity
Glycosides							Drug interaction
H_2_ blockers, antacids, and scralfate						Dec B	
Halofantrine						Contraindicated	
Hydantoins, (phenytoin, Dilantin)		Dec B		Inc A, Dec B	Inc A, Dec B	Inc A, Dec B	
Isoniazid							
Fluconazole							Drug Interaction
Itraconazole							Drug Interaction
Ketoconazole							Drug Interaction
Long acting barbitunates					Contraindicated		
Lovastatin/simvastatin					Inc A		
Methadone					Inc A		
Midazolam/triazolam, po				Inc A	Inc A	Inc A	
Nevirapine		Dec B					
Nifedipine			Inc A				
Oral anticoagulants				Inc A	Inc A		
Oral hypoglycemics (tolbutamide, glipizide, and glyburide)				Inc A	Inc A		
Pentamidine							Inc nephrotoxicity
Pimozide					Contraindicated Inc A	Contraindicated Inc A	
Protease inhibitors					Inc A		
Proton pump inhibitors					Dec B, Inc A		
Quinidine					Contraindicated	Contraindicated	
Ritonavir (400 mg q 12 h)					Contraindicated		
Rifampin/rifabutin		Dec B		Inc A, Dec B	Contraindicated Inc A, Dec B	Inc A, Dec B	
Sirolimus			Inc A		Contraindicated, Inc B		
St. John's Wort					Contraindicated, Dec B		
Tacrolimus		Dec A		Inc A, with toxicity	Inc A, with toxicity	Inc A, with toxicity	
Terfenadine				Contraindication		Contraindicated	
Theophyllines				Inc A			
Thiazide diuretics							Drug interaction
Trazodone							
Warfarin				Inc A			
Zidovudine				Inc A			Drug interaction

**Table:** (b)

Agent	Major side effects	Drug interactions	Cytochrome P450 interactions
Polyenes		The following are applicable to all polyenes	

(1) Amphotericin B	Nephrotoxicity, infusion-related reaction, pain at the site of injection, phlebitis, thrombophlebitis, cardiopulmonary (cardiac arrest, hypotension, tachypnea, and arrthmia) anemia, thrombocytopenia, leukopenia, coagulation defect, anorexia, nausea, diarrhea, generalized pain, muscle, joint pain, headache, anaphylactic reaction, bronchospasm, wheezing, rash, acute liver failure, hepatitis, jaundice, convulsion, and hearing loss	Antineoplastic agents, corticosteroids and corticotropin, digitalis glycosides, flucytosine, azoles, other nephrotoxic medications, skeletal muscle relaxants, and leucocyte transfusion	No interaction in the p450 pathway

(2) AmBisome	Infusion related reaction, renal toxicity, chest pain, hypotension, tachycardia, diarrhea, nausea, vomiting, abdominal pain, bilirubinemia, liver enzymes elevation, hypokalemia, hypomagnesemia, anxiety, headache, lung disorder, pleural effusion, and rash	Same as above	No interaction in the p450 pathway

(3) Amphotericin B colloidal dispersion (ABCD)	Infusion-related reaction, renal toxicity, hypotension, tachycardia, abdominal pain, hypokalemia, diarrhea, nausea vomiting, rash, dyspnea, asthma, confusion, and dizziness	Antineoplastic agents, corticosteroids and corticotropin, digitalis glycosides, and azoles	No interaction in the p450 pathway

(4) Abelcet (ABLC)	Infusion-related reactions, increased serum creatinine, cardiopulmonary (hypotension, tachypnea, arrythmia, pleural effusion, anaphylactic reaction (bronchospasm, wheezing, and asthma), rash, acute liver failure, hepatitis, jaundice, nausea vomiting, abdominal pain, headache, renal toxicity dose dependent, muscle, joint pain, convulsion, and tinnitus	Same as above	No interaction in the p450 pathway

Azoles			

(1) Voriconazole	Visual disturbances, hepatic toxicity, arrhythmia, QT prolongation, and infusion-related reaction	Rifampin and rifabutin, ritonavir, St. John's Wort, carbamazepine and long acting barbiturates, cimetidine, macrolide antibiotics (erythromycin), sirolimus, ergot alkaloids, cyclosporine, methadone, tacrolimus, warfarin, oral coumarin, anticoagulants, statins, benzodiazepines, calcium channel blockers, sulfonylureas, vinca alkaloids, prednisolone, mycophenolic acid, rifabutin, efavirenz, phenytoin, omeprazole, oral contraceptives, other HIV protease inhibitors, other nonnucleoside reverse transcriptase inhibitors, and indinavir	CYP2C19, CYP2C9, and CYP3A4

(2) Fluconazole	Hepatotoxicity, anaphylactic reaction, QT prolongation, seizures, dizziness, skin disorders, leukopenia, thrombocytopenia, hypercholesterolemia, hypokalemia, vomiting, abdominal pain, nausea, and diarrhea	Oral hypoglycemics, coumarin-type anticoagulants, theophylline, rifampin, warfarin, phenytoin, cyclosporine, rifabutin, terfenadine, cisapride, tacrolimus, short acting benzodiazepines, astemizole, hydrochlorothiazide, glimepiride, losartan, methadone, losartan, and cyclophosphamide	Metabolized by cytochrome P450 systems, CYP2C19, CYP2C9, and CYP3A4

(3) Itraconazole	Hepatotoxicity, cardiac dysrhythmias, nausea, diarrhea, vomiting, hypokalemia, bilirubinemia, and rash	Digoxin, dofetilide, quinidine, disopyramide, carbamazepine, nevirapine, rifabutin, busulfan, docetaxel, vinca alkaloids, pimozide, alprazolam, diazepam, midazolam, triazolam, dihydropyridines, verapamil, cisapride, atorvastatin, cerivastatin, lovastatin, simvastatin, cyclosporin, tacrolimus, sirolimus, oral hypoglycemics, indinavir, ritonavir, saquinavir, levacetylmethadol, ergot alkaloids, halofantrine, alfentanil, buspirone, corticosteroids, budesonide, trimetrexate, cilostazol, eletriptan, warfarin, carbamazepine, phenobarbital, phenytoin, isoniazid, rifabutin, rifampine, and erythromycin,	Potent CYP3A4 isoenzyme inhibitor, CYP2C9

(4) Posaconazole	Hepatotoxicity, diarrhea, nausea, vomiting, abdominal pain, hypokalemia, thrombocytopenia, fever, rigors, headache, fatigue, hypotension, hypertension, anemia, neutropenia, and rash	Rifabutin, phenytoin, cimetidine, cyclosporin, tacrolimus, midazolam, terfenadine, astemizole, pimozide, cisapride, and quinidine	Inhibitor of CYP3A4

Echinocandins			

(1) Anidulafungin	Histamine-mediated symptoms, dyspnea, and hypotension	Cyclosporin (no dose adjustment needed)	Not an inducer, inhibitor, or substrate of the P450 system

(2) Caspofungin	Histamine-mediated symptoms, anaphylactic reaction, hyperbilirubinemia, and rash	Tacrolimus, cyclosporin, rifampin, efavirenz, nevirapine, phenytoin, dexamethasone, and carbamazeine	Is a poor substrate for cytochrome P450 enzymes

(3) Micafungin	Histamine-mediated symptoms (rash, pruritus, facial swelling, and vasodilation), diarrhea, nausea, vomiting, pyrexia, hypokalemia, thrombocytopenia, headache, hepatocellular damage, delirium, skin disorders (skin necrosis, urticaria), convulsions, and arthralgia	Sirolimus (AUC increased by 21%, no effect on micafungin), nifedipine (AUC increased by 18%, no effect on micafungin), and itraconazole (AUC increased by 22%, no effect on micafungin)	Micafungin is not an inducer or inhibitor of P-glycoprotein, but is an inducer of CYP3A4

**Table 4:** Current contraindications and FDA warnings for each antifungal agent available.

Drug	Contraindications	Warnings
Amphotericin B deoxycholate	Hypersensitivity to amphotericin B	Anaphylaxis

Lipid formulations of AMB	Hypersensitivity to amphotericin B	Anaphylaxis

Fluconazole	Hypersensitivity to fluconazole	Hepatic injury, anaphylaxis, and dermatologic

Itraconazole	Terfenadine, astemizole, dofetilide, pimozide, quinidine, oral midazolam, triazolam, cisapride, and statins should also be discontinued during therapy	Black box for terfenadine and congestive Heart failure see contraindications

Posaconazole	Hypersensitivity to the active substance or excipients, ergot alkaloids, coadministration with 3A4 substrates (terfenadine, astemizole, cisapride, pimozide, halofantrine, and quinidine)	Hypersensitivity, hepatic toxicity, recommended monitoring of hepatic function (LFTs), cyclosporine, tacrolimus, and sirolimus

Voriconazole	Hypersensitivity to voriconazole, CYP3A4 inhibitors (terfenadine, astemizole, cisapride, pimozide, and quinidine), sirolimus, rifampin, carbamezapine, long acting barbiturates, ritonavir, efavirenz, rifabutin, and ergot alkaloids (ergotamine and dihydroergotamine)	Visual disturbances, hepatic toxicity, recommended monitoring of LFTs and bilirubin, pregnancy category D, and galactose intolerance

Anidulafungin	Hypersensitivity to anidulafungin or other echinocandins	None

Caspofungin	Hypersensitivity to caspofungin or other echinocandins	Elevated liver enzymes with cyclosporine

Micafungin	Hypersensitivity to micafungin or other echinocandins	Hypersensitivity, hematological effects (hemolysis, hemolytic anemia, and hemoglobinuria), hepatic effects (abnormal LFTs, hepatic dysfunction, hepatitis, and hepatic failure), and renal effects (elevations of BUN and creatinine, renal dysfunction, and acute renal failure)

**Table 5:** Pharmacokinetic parameters of the major echinocandins in clinical use*.

Agent	*C*_max⁡_	*t*_1/2_	*V*_*d*_	AUC	*C*_*t*_	FeU	FeS
Caspofungin	7.64	10	0.4	88–115	0.15	1.40%	35%
Micafungin	4.95	14	0.23	111	0.19	0.70%	40%
Anidulafungin	2.07–3.5	25	0.5	44–53	0.26	<1%	30%, <10% unchanged

*Dose 50 mg single dose, *C*_max⁡_: maximum concentration, *t*_1/2_: elimination half-life, *V*_*d*_: volume of distribution, AUC: area under the plasma concentration and time curve, *C*_*t*_: total clearance, FeU: fraction excreted in urine, FeS: fraction excreted in stool.

**Table:** (a) Experience in patients with prexisting renal disease

Agent	Effects on the kidney	Dosing modifications for preexisting renal disease
(1) Amphotericin B	Nephrotoxic-elevation of BUN, creatinine	Sodium loading to ameliorate toxicity

(2) AmBisome	Nephrotoxic	Used in pt with pre-existing renal impairment

(3) ABCD	Nephrotoxic	

(4) ABLC	Dose-limited renal toxicity	

(5) Voriconazole	SBECD component of iv formulation	No adjustment for oral vori in pts with mild-to-severe renal impairment
	associated with renal toxicity	I.V. should be avoided if creatinine clearance <30 mL/min

(6) Fluconazole		50–400 mg creatinine clearance >50–100% creatinine clearance <50 (no dialysis)-adm. 50% of dose
		regular dialysis-admin 100% of dose after each dialysis

(7) Itraconazole	SBECD component of iv formulation	I.V. should not be used if creatinine clearance <30 mL/min
	associated with renal toxicity	

(8) Posaconazole		No dose adjustment for mild-to-moderate severe-monitor for breakthrough IFI

(9) Anidulafungin	None	No dose adjustment, not dialyzable

(10) Caspofungin	None	No dose adjustment, not dialyzable

(11) Micafungin	None	No dose adjustment

**Table:** (b) Experience in individuals with pre-existing hepatic disease

Antifungal	Normal Patients	Mild	Moderate	Severe	Effects on the liver
		(Child-Pugh 5-6)	(Child-Pugh 7–9)	(Child-Pugh >9)	
Anidulafungin	200 mg loading dose on day 1 followed by 100 mg once/day	200 mg loading dose on day 1 followed by 100 mg once/day	200 mg loading dose on day 1 followed by 100 mg once/day	200 mg loading dose on day 1 followed by 100 mg once/day	None

Micafungin	100 mg once/day	100 mg once/day	100 mg once/day	Not studied	None

Caspofungin	70 mg loading dose on day 1 followed by 50 mg once/day	70 mg loading dose on day 1 followed by 50 mg once/day	70 mg loading dose on day 1 followed by 35 mg once/day	Not studied	None

Fluconazole	Loading dose of 2x the daily dose, then up to 400 mg daily	No dosage adjustments initially, monitor LFTs in patients for worsening hepatic function			Hepatotoxic

Itraconazole	200 mg q12 IV 100–200 mg q12 po-solution	No studies have been conducted looking at patients with hepatic impairment, use with caution			Hepatotoxic, prolonged elimination half-life in cirrhotic patients (meds metabolized by CYP3A4)

Voriconazole	6 mg/kg IV q12h for the first 24 hours loading dose followed by 3-4 mg/kg IV q12h maintenance dose then 200 mg q12h oral	6 mg/kg IV q12h for the first 24 hours loading dose followed by 1.5–2 mg/kg IV q12h maintenance dose	6 mg/kg IV q12h for the first 24 hours loading dose followed by 1.5–2 mg/kg IV q12h maintenance dose	Not studied	Hepatotoxic

Posaconazole	Oral Suspension 200 mg (5 mL) three times a day with a full meal or liquid nutritional supplement, monitoring of LFT's is recommended	Data was not sufficient to determine dosing, should be used with caution			Mild-to-moderate elevation of liver enzymes, bilirubin-generally reversible

Amphotericin B deoxycholate	0.6 to 1 mg/kg/day	Data was not sufficient to determine dosing			Elevation of liver enzymes

Ampho B lipid complex (Abelcet)	5 mg/kg/day	Data was not sufficient to determine dosing			Elevation of liver enzymes

Ampho B colloidal dispersion (Amphotec)	3-4 mg/kg/day can be increased up to 6 mg/kg/day	Data was not sufficient to determine dosing			Elevation of liver enzymes

Ampho B liposomal (AmBisome)	3-4 mg/kg/day can be increased up to 6 mg/kg/day	Data was not sufficient to determine dosing			Elevation of liver enzymes
